# Circulating miR-449a predicts survival outcome for colorectal cancer following curative resection: An observational study

**DOI:** 10.1097/MD.0000000000025022

**Published:** 2021-04-16

**Authors:** Dengke Fu, Yang Chen, Dongkui Xu

**Affiliations:** a Department of Oncology, Chuiyangliu Hospital Affiliated to Tsinghua University; b Department of VIP Medical Services, National Cancer Center/National Clinical Research Center for Cancer/Cancer Hospital, Chinese Academy of Medical Sciences and Peking Union Medical College, Beijing, PR China.

**Keywords:** colorectal cancer, microRNA -449a, prognosis

## Abstract

Previous studies showed that microRNA (miR)-449a may function as a tumor suppressor. However, the expression pattern and value of circulating miR-449a in colorectal cancer (CRC) remain unclear. Therefore, the purpose of this study was to measure circulating miR-449a level of CRC patients and evaluate its value for predicting prognosis.

Plasma samples of 343 consecutive CRC patients and 162 healthy controls were obtained. Circulating miR-449a levels were measured by using real-time quantitative reverse transcription polymerase chain reactions. All enrolled patients were followed up in a regular interval after surgery. The clinical data and survival outcome of all 343 patients were collected. The correlation between circulating miR-449a level and survival outcomes was analyzed by univariate and multivariate analysis.

Circulating miR-449a level in CRC patients was significantly decreased (*P* < .05) comparing with healthy controls. Low miR-449a was significantly associated with CEA and CA19-9 level (both *P* < .05). Furthermore, patients with a decreased miR-449a level had a lower 5-years overall survival (OS) rate than those with a high miR-449a (67.4% vs 76.9%, *P* = .03). Low circulating miR-449a level also been demonstrated as an independent risk factor for CRC in multivariate COX analysis (HR, 2.56; 95%CI: 1.15–8.63; *P* < .05).

Circulating miR-449a was significantly decreased in CRC patients and closely related to poor prognosis, suggesting that miR-449a might can be used as a useful diagnostic and prognostic marker for CRC.

## 1 Introduction

Colorectal cancer (CRC) is one of the most frequent diagnostic and lethal gastrointestinal malignancies in China.^[1,2]^ Although earlier diagnosis and advanced therapeutic regimens have increased survival outcome of patients with CRC over the past decades, a large part of patients received curative surgery subsequently suffer from recurrence, thus had an unsatisfactory prognosis.^[3,4]^ Moreover, for patients experienced recurrent, chemotherapy and adjuvant therapies may produce adverse side effects and substantial financial costs, thereby exert adverse effect on quality of life, as well as economic burden.^[5,6]^ In addition, CRC patient population always differ greatly in physiological-psychological status and treatment responses. Therefore, there is a clear requirement for biomarkers that will facilitate efficient and effective early risk stratification of patients with CRC.^[7]^ Many well-established models or biomarkers for the risk stratification and prognosis prediction of CRC patients has been reported.^[8,9]^ However, most of such models or biomarkers mainly depend on postoperative pathological evaluations, which had no significance in guiding preoperative neo-adjuvant therapies.^[10–12]^ Therefore, pretreatment biomarkers with characteristics of feasible, effective, and noninvasive, for prognosis prediction and risk stratification, are urgently required.

MicroRNAs (miRNAs) are a subset of small noncoding RNAs that post-transcriptionally regulate gene expression by specifically inhibiting target messenger RNAs (mRNAs).^[13]^ Dysregulated miRNAs expression can be frequently observed in various human malignancies.^[14–19]^ Most previous studies concerning miRNA biomarkers mainly relied on analysis of tissue specimens, while recently studies have showed that circulating miRNAs could maintain measurable and stable status in serum or plasma samples.^[20,21]^ Therefore, recently, expression characteristics of circulating miRNAs in cancers have been increasingly evaluated.^[22–24]^

MiRNA-449a is considered as a tumor suppressive miRNA, with a remarkably decreased expression level in cancers.^[25]^ It has been showed that downregulated miR-449a expression was related advanced clinical stage and poor histological differentiation of CRC.^[26]^ In perspective of underlying mechanism, miR-449a inhibits the growth and metastasis of CRC cells by targeting Notch-1, HADC-1, and SATB2.^[27,28]^ However, no studies have investigated the value of circulating miR-449a level in CRC patients. In current study, we focused on circulating level of miRNA-449a in CRC patients and evaluate its value for postoperative prognosis.

## 2 Materials and methods

### 2.1 Ethics approval, study design, and subjects

The study protocol was approved by the Ethics Committee of Chinese Academy of Medical Sciences and Peking Union Medical College and conducted in accordance with the tenets of the Declaration of Helsinki and relevant guidelines. Written informed consent was required for each subject for the use of blood samples and clinical data in this study.

This study is a retrospective analysis of prospectively comprehensive miRNA microarray analyses database. Data of all CRC patients received curative resection between May 2017 and July 2019 at the Cancer Hospital of Chinese Academy of Medical Sciences were retrospectively obtained. All enrolled CRC patients had pathologically confirmed localized adenocarcinoma and did not receive any anticancer therapies. Exclusion criteria included preoperative acute and severe comorbidity, contraindication of curative resection, unavailable and incomplete clinical and pathological information and survival expectancy less than 24 wk. All patients received curative surgery and appropriate adjuvant treatments according to 2015 NCCN Colorectal Cancer Practice Guidelines.^[29]^ Clinical and pathological information of all enrolled CRC patients were obtained and checked. Tumor stage was evaluated according to tumor-node-metastasis (TNM) staging system of the Seventh Edition of the Union for International Cancer Control. Histologic grade was assessed based on the World Health Organization criteria. Age and sex-matched healthy volunteers without history of malignant disorders or congenital diseases and in health status based on routine physical examinations were enrolled as the control group.

After discharging, following-up was conducted for each patient though clinical visiting or telephone in a regular style, which lasted from the surgery time to either death or June 2020. The survival was evaluated as the primary outcome, presenting as 5-years overall survival (OS) rate.

### 2.2 Sample preparation and RNA isolation

Almost 5 ml sterile peripheral blood was collected from each enrolled participant. Plasma was extracted by cell-free nucleic acid isolation using a 2-spin protocol (16,000 × g for 10 minutes at 4°C and 1600 × g for 10 minutes at 4°C), then transferred to a RNase/DNase-free tubes sored at − 80°C until for use. Total RNA extraction was performed by using TRIzol reagent (Invitrogen, Carlsbad, California) according to the corresponding instructions. Furthermore, the concentration and quality of RNA were measured by using a NanoDrop 2000 spectrophotometer (Nanodrop Technologies).

### 2.3 Quantification of miRNA by using real-time quantitative reverse transcription polymerase chain reactions

Total RNA sample was used to reversely transcribe miRNAs to a strand cDNA using a TaqMan MicroRNA reverse transcription kit (Applied Biosystems, Foster City, CA), and then was quantified by using the QuantMir RT Kit (System Biosciences, Mountain View, CA) in ABI 7900 Sequence Detection System (Applied Biosystems) with miR-449a und U6 primers (Applied Biosystems). The expression levels of each miRNAs were normalized against U6 expression. The relative expression levels of serum miR-449a was quantitatively analyzed by using the 2–^ΔΔCT^ method.

### 2.4 Statistical analysis

The SPSS 20.0 software (IBM) was used to analyses all obtained data. *P* < .05 (2 sided) was considered to indicate a statistically significant difference. The Chi Squared test and Fisher exact probability method was used to evaluate the relationship between circulating miR-449a levels and clinico-pathological variables. Receiver operating characteristic curve (ROC) analysis was adopted to analyze value of the circulating miRNA-449a levels in detecting CRC. The Kaplan–Meier survival curve method and log-rank test were conducted to compare the survival rate between groups. Multivariate Cox hazard regression model was employed to confirm the independent prognostic factors for CRC.

## 3 Results

### 3.1 Circulating miR-449a level in CRC patients

**Figure 1:**
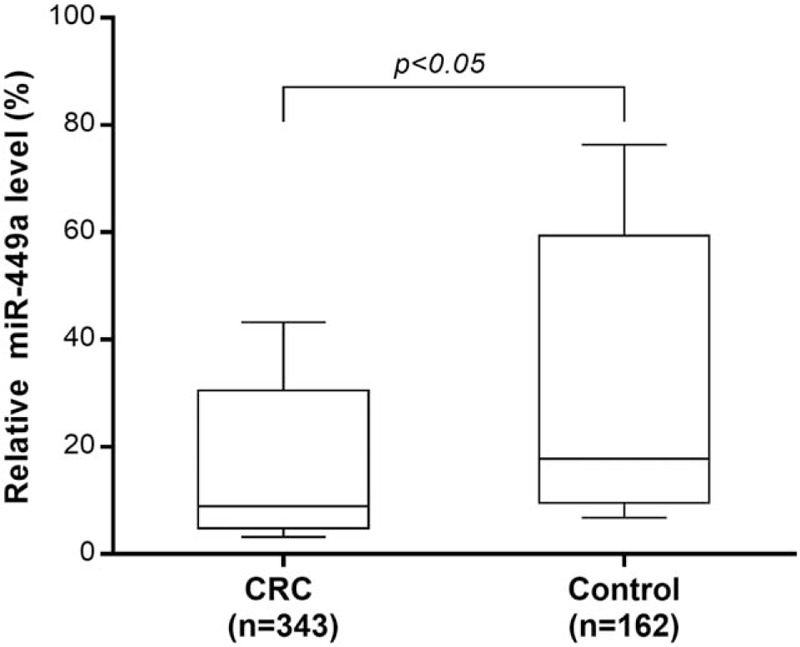
Serum miR-449a level in colorectal cancer patients and healthy controls. The circulating miR-449a levels of 343 colorectal cancer patients was significant lower than that of 162 age-matched healthy volunteers (*P* < .05).

**Figure 2:**
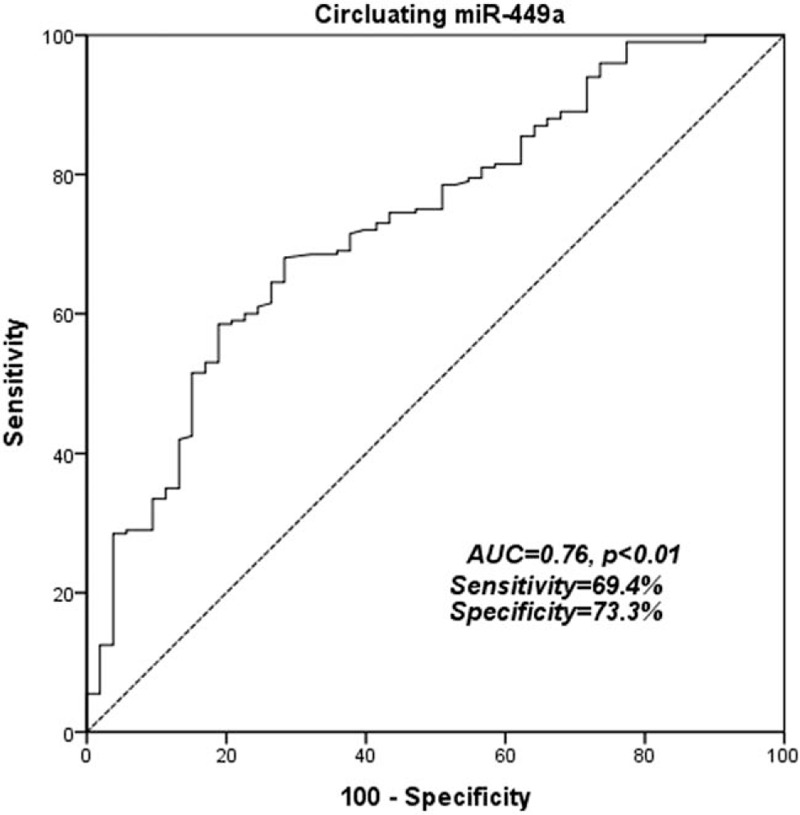
Receiver-operator characteristic curve for CRC detection. ROC analysis showed an area under the curve of 0.76 for miR-449a with a 95% confidence interval between 0.58 and 0.94, *P* < .01.

MiR-449a expression can be found in plasma sample of each participant. CRC patients had a significant lower mean serum miR-449a level than that of healthy subjects (*P* < .05, see Fig. 1). Furthermore, in ROC curve, circulating miR-449a level can significantly distinguishing CRC patients from healthy subjects with a sensitivity of 69.4% and a specificity of 73.3%, and an area under the curve of 0.76 (95%CI:0.58–0.94, *P* < .05, see Fig. 2).

### 3.2 Correlation between circulating miR-449a and clinicopathological variables

In this study, Chi-Squared test was applied to evaluate the association between circulating miR-449a levels and all obtained clinicopathological characteristics, which showed that the circulating level of miR-449a was significantly associated with carcinoembryonic antigen (CEA) and carbohydrate antigen 19–9 (CA19–9) (both *P* < .05). However, no obvious differences between high and low miR-449a level groups in terms of the other variables was found, including gender, age, site and size of tumor, pathological stage and TNM stage (see Table 1).

**Table 1:** Correlation between circulating miR-449a level and clinic variables of CRC patients.

	Circulating miR-449a		
Characteristic	High (n = 117)	Low (n = 226)	*P*
Age (yr)			.40
≥60	56 (47.9%)	97 (42.9%)	
<60	61 (52.1%)	129 (57.1%)	
Gender			.64
Male	74 (63.2%)	137 (60.6%)	
Female	43 (36.8%)	89 (39.4%)	
Tumor site			.07
Colon	51 (43.6%)	76 (33.6%)	
Rectum	66 (56.4%)	150 (66.4%)	
Tumor size (cm)			.99
≥5	43 (36.8%)	83 (36.7%)	
<5	74 (63.2%)	143 (63.3%)	
T stage			.10
T1+T2	74 (63.2%)	122 (54.0%)	
T3	43 (36.8%)	104 (46.0%)	
Nodes involvement			.74
N0	46 (39.3%)	93 (41.2%)	
N1	71 (60.7%)	133 (58.8%)	
Clinical stage (TNM)			.53
I+II	79 (67.5%)	145 (64.2%)	
III	38 (32.5%)	81 (35.8%)	
Pathological differentiation			.25
Well/Moderate	66 (56.4%)	142 (62.8%)	
Poor	51 (43.6%)	84 (37.2%)	
CEA (ng/ml)			<.01
≥5	10 (8.5%)	80 (35.4%)	
<5	107 (91.5%)	146 (64.6%)	
CA19-9 (ng/ml)			<.01
≥38	50 (42.7%)	44 (19.5%)	
<38	67 (57.3%)	182 (80.5%)	

### 3.3 Prognostic significance of circulating miR-449a level in CRC patients

**Figure 3:**
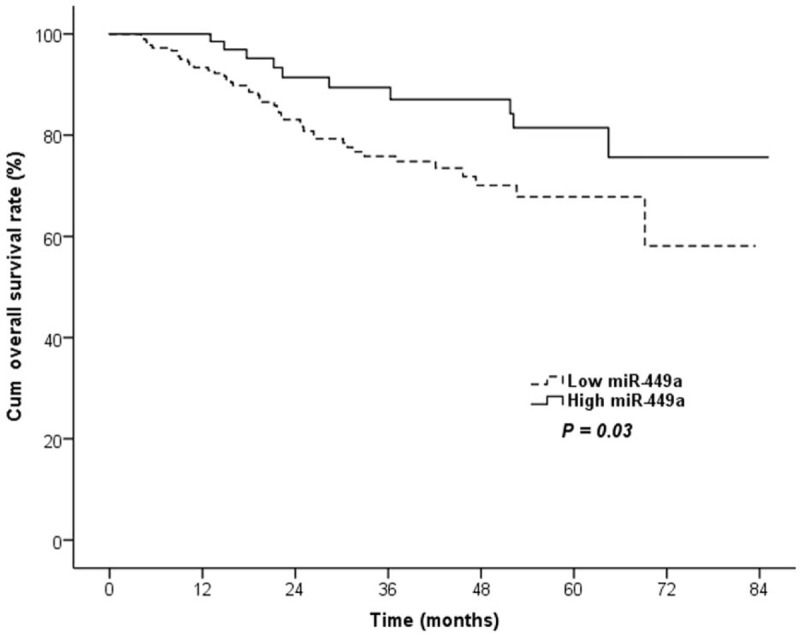
Lower circulating miR-449a level was associated with worse prognosis for colorectal cancer. The prognostic analysis revealed that a low miR-449a level was significantly associated with a worse 5-year overall survival rate (*P* = .03).

All patients were followed up with a median duration of 35.6 months (range, 6.8–78.5months). Among the whole CRC group completing following-up, 79 (21.8%) patients had tumor-special death. The univariate analysis showed that patients with decreased circulating level of miR-449a had a significantly lower 5-year OS rate than those with high miR-449a (67.4% vs. 76.9%, *P* = .03, Table 2., Fig. 3.).

**Table 2:** The survival analysis of CRC patients.

	Univariate			Multivariate		
	n	5-yr OS rate	*P*	HR	95%CI	*P*
Age (yr)			.88			
≥60	153	66.8%				
<60	190	72.3%				
Gender			. 24			
Male	211	72.5%				
Female	132	71.8%				
Tumor site			.41			
Colon	127	70.6%				
Rectum	216	69.5%				
Tumor size (cm)			.16			
≥5	126	69.3%				
<5	217	72.8%				
Tumor invasion depth			.56			
T1+T2	181	75.7%				
T3+T4	162	69.6%				
Lymph node involvement			.01			
N0	139	74.8%				
N1	204	67.3%				
Clinical Stage			.03	4.37	3.98–12.33	<.01
I+II	150	75.3%				
III	203	64.2%				
Pathological differentiation			.03	1.28	1.01–6.63	.01
Well/Moderate	208	76.2%				
Poor	135	65.1%				
MiR-449a			.03	2.56	1.15–8.63	<.01
low	226	67.4%				
high	117	76.9%				
CEA (ng/ml)			<.01			
≥5	90	65.4%				
<5	253	75.8%				
CA19–9 (ng/ml)			<.01	2.15	1.02–8.88	.02
≥38	94	64.3%				
<38	249	76.1%				

The multivariate analysis by using COX regression model was applied to confirm independent risk factors for CRC patients, which showed that a decreased circulating miR-449a level (*P* < .01; HR, 2.56; 95% confidence interval [CI]: 1.15–8.63) was an independent risk factor for patients with CRC independent of conventional prognostic indictors, such as clinical TNM stage (*P* < .01; hazard ratio, 4.37; 95%CI: 3.98–12.33) and pathological differentiation (*P* = .01; hazard ratio, 1.28; 95%CI: 1.01–6.63) (see Table 2).

## 4 Discussion

In this study, we evaluated the preoperative circulating expression pattern of tumor suppressor miR-449ain CRC patients and found that miR-449a level was significantly decreased in CRC patients while comparing with healthy controls. Moreover, circulating miR-449a can be used as an indicator for discriminating CRC patients from healthy. Moreover, we also analyzed the potential significance of preoperative circulating miR-449a as a candidate prognostic indictor for CRC patients, and showed decreased miR-449a level was significantly associated with poor OS outcome. We also suggested that there was a significant correlation between a low serum miR-449a levels and the levels of classical tumor markers in CRC patients, such as CEA and CA19–9. According to results mentioned above, we suggested that miR-449a can be applied in diagnosis and risk stratification of patients with CRC, and preoperative circulating miR-449a also can sever as a promising serum prognostic marker for CRC patients.

MiR-449a severs as a member of the miR-449 family which consists of similar sequences and structures as the miR-34 family and exerts critical roles in the development of several cancer types. The tissue expression level of miR-449a is decreased in CRC cancer and serves as a tumor suppressor.^[26]^ Previous studies conducting miR-449a knockdown in mice model showed that the deficiency of miR-449a was associated with tumorigenesis of CRC.^[30]^ Furthermore, miR-449a expression was associated with down-regulation of several oncogenes, such as HDAC1, transforming growth factor-β (TGFB), a disintegrin and metalloproteinase domain-containing protein 10 (ADAM10) and mitogen-activated protein kinase 1 (MAPK1), special ATrich sequence-binding protein 2 (SATB2).^[31–33]^ ISHIKAWA et al also showed that miR-449a level in tumor tissue might be a prognostic biomarker for CRC and miR-449a might regulate HDAC1 expression.^[26]^ In present study, we also showed that circulating miR-449a was highly decreased in CRC patients and associated with poor prognosis in CRC patients.

Previous studies involving miR-449a showed that colon cancer cell lines had a higher expression of miR-449 than the normal colon tissues.^[34]^ The current study directly evaluated the serum expression of miR-449a in colorectal carcinoma patients. To the best of our knowledge, the present study is the first to report the elevated serum expression of miR-449a in colorectal carcinoma patients. Compared to pathological diagnosis by tissue sampling, serum samples are easier to obtain and exert noninvasive to patients. However, the clear and definite function and underlying mechanism of circulating miR-449a in cancer have not yet been fully revealed. Previous studies have reported several mechanisms of circulating miRNAs releasing, including passive leakage and active secretion. Moreover, circulating miRNAs from cancer cells can induce tumorigenesis in the recipient cells.^[35–37]^ Previous studies showed that circulating levels of miR-18a-5p, miR-21–5p, miR-29a-5p, miR-92a-5p, miR-143–5p and miR-378–5p were significantly decreased in patients with CRC.^[38]^ ISHIKAWA et al reported that the miR-449a level in tumor tissue might be useful as a prognostic indicator for patients with gastric cancer and associated with CEA and CA19–9 levels.^[39]^ In this study, we also showed similar results that circulating miR-449a level was significantly associated with CEA and CA19–9 in CRC patients. Chen et al also reported that increased miR-449a expression in colorectal carcinoma tissues was inversely correlated with serum CEA.^[40]^ Therefore, serum miR-449a level might be a new treatment target for CRC patients.

The present study firstly showed that the circulating miR-449a level in CRC patients, which can be used as both a serum prognostic biomarker and a novel target for CRC treatments. But there were several limitations in present study. This study has retrospective design and was performed in a single center. A large-scale, multicenter prospective study is required to furtherly confirm these results. Moreover, the roles of miR-449a in growth of colorectal cancer and underlying mechanisms have not yet been fully understand. Further experiments need to be performed to analyze the mechanisms of miR-449a in carcinogenesis.

## 5 Conclusions

In conclusion, circulating miR-449a levels were decreased in CRC patients. Moreover, low circulating expression of miR-449a were positively associated with poor prognosis of CRC, indicating that miR-449a may act as a tumor suppressor in CRC. Circulating miR-449a might not only serve as a diagnostic and prognostic indictor of CRC, but also as a potential novel target for CRC treatment.

## Author contributions

**Conceptualization:** Dongkui Xu.

**Data curation:** Dengke Fu, Yang Chen, Dongkui Xu.

**Formal analysis:** Dengke Fu, Yang Chen.

**Funding acquisition:** Dengke Fu, Dongkui Xu.

**Investigation:** Yang Chen, Dongkui Xu.

**Methodology:** Yang Chen, Dongkui Xu.

**Resources:** Dengke Fu, Yang Chen.

**Software:** Yang Chen.

**Validation:** Dengke Fu.

**Visualization:** Dongkui Xu.

**Writing – original draft:** Dengke Fu, Yang Chen.

**Writing – review & editing:** Dengke Fu, Yang Chen, Dongkui Xu.

## References

[1] Siegel RL Miller KD Fedewa SA. Colorectal cancer statistics, 2017. CA Cancer J Clin 2017;67:177–93. 28248415 10.3322/caac.21395

[2] Chen W Zheng R Baade PD. Cancer statistics in China, 2015. CA Cancer J Clin 2016;66:115–32. 26808342 10.3322/caac.21338

[3] Fleming CA Ullah MF Chang KH. Propensity score-matched analysis comparing laparoscopic to robotic surgery for colorectal cancer shows comparable clinical and oncological outcomes. J Robot Surg 2020. 10.1007/s11701-020-01116-0 32643095

[4] Lugli A Zlobec I. The battle for prognosis at the invasive front of colorectal cancer. EBioMedicine 2020;58:102918. 32711249 10.1016/j.ebiom.2020.102918 PMC7387774

[5] Kataoka S Nishikawa Y Funakoshi T. Long-term survival and renal dysfunction in a patient with recurrent colorectal cancer treated with Bevacizumab. Clin J Gastroenterol 2020;13:316–9. 31707696 10.1007/s12328-019-01060-z

[6] Matsuoka H Ogata Y Nakamura M. An observational study of team management approach for CapeOX therapy in patients with advanced and recurrent colorectal cancer: SMILE Study (The Study of Metastatic colorectal cancer to investigate the Impact of Learning Effect). J Anus Rectum Colon 2020;4:79–84. 32346646 10.23922/jarc.2019-020 PMC7186009

[7] Nagtegaal ID Quirke P Schmoll HJ. Has the new TNM classification for colorectal cancer improved care? Nature reviews. Clin Oncol 2011;9:119–23. 10.1038/nrclinonc.2011.157 22009076

[8] Han J Zhang X Zhang AD. [Impact of primary tumor site on the prognosis in different stage colorectal cancer patients after radical resection]. Zhonghua wai ke za zhi [Chin J Surg] 2018;56:68–73. 10.3760/cma.j.issn.0529-5815.2018.01.015 29325357

[9] Li W Zhang G Wang HL. Analysis of expression of cyclin E, p27kip1 and Ki67 protein in colorectal cancer tissues and its value for diagnosis, treatment and prognosis of disease. Eur Rev Med Pharmacol Sci 2016;20:4874–9. 27981549

[10] Van Schaeybroeck S Allen WL Turkington RC. Implementing prognostic and predictive biomarkers in CRC clinical trials. Nature reviews. Clin Oncol 2011;8:222–32. 10.1038/nrclinonc.2011.15 21321566

[11] Quirke P Williams GT Ectors N. The future of the TNM staging system in colorectal cancer: time for a debate? Lancet Oncol 2007;8:651–7. 17613427 10.1016/S1470-2045(07)70205-X

[12] Lorenc Z Waniczek D Lorenc-Podgorska K. Profile of expression of Genes Encoding Matrix Metallopeptidase 9 (MMP9), Matrix Metallopeptidase 28 (MMP28) and TIMP Metallopeptidase Inhibitor 1 (TIMP1) in colorectal cancer: assessment of the role in diagnosis and prognostication. Med Sci Monit 2017;23:1305–11. 28293015 10.12659/MSM.901593 PMC5363457

[13] Mohr AM Mott JL. Overview of microRNA biology. Semin Liver Dis 2015;35:03–11. 10.1055/s-0034-1397344 PMC4797991 25632930

[14] Zhang Y Guo L Li Y. MicroRNA-494 promotes cancer progression and targets adenomatous polyposis coli in colorectal cancer. Mol Cancer 2018;17:01. 10.1186/s12943-017-0753-1 PMC5755155 29304823

[15] Zabaglia LM Bartolomeu NC Dos Santos MP. Decreased microRNA miR-181c expression associated with gastric cancer. J Gastrointest Cancer 2017;49:97–101. 10.1007/s12029-017-0042-7 29243018

[16] Polasik A Tzschaschel M Schochter F. Circulating tumour cells, circulating tumour DNA and circulating microRNA in metastatic breast carcinoma-what is the role of liquid biopsy in breast cancer? Geburtshilfe Frauenheilkd 2017;77:1291–8. 29269956 10.1055/s-0043-122884 PMC5734937

[17] Qin C Zhao Y Gong C. MicroRNA-154/ADAM9 axis inhibits the proliferation, migration and invasion of breast cancer cells. Oncol Lett 2017;14:6969–75. 29163713 10.3892/ol.2017.7021 PMC5686518

[18] Li H Jiang X Niu X. Long non-coding RNA reprogramming (ROR) promotes cell proliferation in colorectal cancer via affecting P53. Med Sci Monit 2017;23:919–28. 28216611 10.12659/MSM.903462 PMC5330205

[19] Sun X Yuan W Hao F. Promoter methylation of RASSF1A indicates prognosis for patients with Stage II and III colorectal cancer treated with oxaliplatin-based chemotherapy. Med Sci Monit 2017;23:5389–95. 29128865 10.12659/MSM.903927 PMC5697441

[20] Niedźwiecki S Piekarski J Szymańska B. Serum levels of circulating miRNA-21, miRNA-10b and miRNA-200c in triple-negative breast cancer patients. Ginekol Pol 2018;89:415–20. 30215459 10.5603/GP.a2018.0071

[21] Zou X Wei J Huang Z. Identification of a six-miRNA panel in serum benefiting pancreatic cancer diagnosis. Cancer Med 2019;8:2810–22. 31006985 10.1002/cam4.2145 PMC6558458

[22] Kosaka N Iguchi H Ochiya T. Circulating microRNA in body fluid: a new potential biomarker for cancer diagnosis and prognosis. Cancer Sci 2010;101:2087–92. 20624164 10.1111/j.1349-7006.2010.01650.x PMC11159200

[23] Zhang J Wang T Zhang Y. Upregulation of serum miR-494 predicts poor prognosis in non-small cell lung cancer patients. Cancer Biomark 2017;21:763–8. 10.3233/CBM-170337 PMC13078321 29286916

[24] Shi M Jiang Y Yang L. Decreased levels of serum exosomal miR-638 predict poor prognosis in hepatocellular carcinoma. J Cell Biochem 2017;119:4711–6. 10.1002/jcb.26650 29278659

[25] Chen J Zhou J Chen X. MiRNA-449a is downregulated in osteosarcoma and promotes cell apoptosis by targeting BCL2. Tumour Biol 2015;36:8221–9. 26002578 10.1007/s13277-015-3568-y

[26] Ishikawa D Takasu C Kashihara H. The significance of microRNA-449a and its potential target HDAC1 in patients with colorectal cancer. Anticancer Res 2019;39:2855–60. 31177123 10.21873/anticanres.13414

[27] Buurman R Gürlevik E Schäffer V. Histone deacetylases activate hepatocyte growth factor signaling by repressing microRNA-449 in hepatocellular carcinoma cells. Gastroenterology 2012;143:811–20.e815. 22641068 10.1053/j.gastro.2012.05.033

[28] Bou Kheir T Futoma-Kazmierczak E Jacobsen A. MiR-449 inhibits cell proliferation and is down-regulated in gastric cancer. Mol Cancer 2011;10:29. 21418558 10.1186/1476-4598-10-29 PMC3070685

[29] Ransohoff DF Sox HC. Clinical practice guidelines for colorectal cancer screening: new recommendations and new challenges. JAMA 2016;315:2529–31. 27304798 10.1001/jama.2016.7990

[30] Niki M Nakajima K Ishikawa D. MicroRNA-449a deficiency promotes colon carcinogenesis. Sci Rep 2017;7:10696. 28878284 10.1038/s41598-017-10500-0 PMC5587792

[31] Noonan EJ Place RF Pookot D. MiR-449a targets HDAC-1 and induces growth arrest in prostate cancer. Oncogene 2009;28:1714–24. 19252524 10.1038/onc.2009.19

[32] Lizé M Pilarski S Dobbelstein M. E2F1-inducible microRNA 449a/b suppresses cell proliferation and promotes apoptosis. Cell Death Differ 2010;17:452–8. 19960022 10.1038/cdd.2009.188

[33] Luo W Huang B Li Z. MicroRNA-449a is downregulated in non-small cell lung cancer and inhibits migration and invasion by targeting c-Met. PLoS One 2013;8:e64759. 23734217 10.1371/journal.pone.0064759 PMC3667122

[34] Guo C Sah JF Beard L. The noncoding RNA, miR-126, suppresses the growth of neoplastic cells by targeting phosphatidylinositol 3-kinase signaling and is frequently lost in colon cancers. Genes Chromosomes Cancer 2008;47:939–46. 18663744 10.1002/gcc.20596 PMC2739997

[35] Chen X Ba Y Ma L. Characterization of microRNAs in serum: a novel class of biomarkers for diagnosis of cancer and other diseases. Cell Res 2008;18:997–1006. 18766170 10.1038/cr.2008.282

[36] Zernecke A Bidzhekov K Noels H. Delivery of microRNA-126 by apoptotic bodies induces CXCL12-dependent vascular protection. Sci Signal 2009;2:ra81. 19996457 10.1126/scisignal.2000610

[37] Wang K Zhang S Weber J. Export of microRNAs and microRNA-protective protein by mammalian cells. Nucleic Acids Res 2010;38:7248–59. 20615901 10.1093/nar/gkq601 PMC2978372

[38] Desmond BJ Dennett ER Danielson KM. Circulating extracellular vesicle microRNA as diagnostic biomarkers in early colorectal cancer-a review. Cancers (Basel) 2019;12. 10.3390/cancers12010052 PMC7016718 31878015

[39] Ishikawa D Yoshikawa K Takasu C. Expression level of microRNA-449a predicts the prognosis of patients with gastric cancer. Anticancer Res 2020;40:239–44. 31892572 10.21873/anticanres.13945

[40] Chen S Dai Y Zhang X. Increased miR-449a expression in colorectal carcinoma tissues is inversely correlated with serum carcinoembryonic antigen. Oncol Lett 2014;7:568–72. 24396489 10.3892/ol.2013.1737 PMC3881925

